# Synthesis, spectroscopic, dielectric, molecular docking and DFT studies of (3E)-3-(4-methylbenzylidene)-3,4-dihydro-2*H*-chromen-2-one: an anticancer agent

**DOI:** 10.1186/s13065-016-0230-8

**Published:** 2017-01-10

**Authors:** T. Beena, L. Sudha, A. Nataraj, V. Balachandran, D. Kannan, M. N. Ponnuswamy

**Affiliations:** 1 Department of Physics, SRM University, Ramapuram, Chennai, 600089 India; 2 Research Department of Physics, A.A. Government Arts College, Musiri, Tiruchirapalli, 621211 India; 3 Department of Organic Chemistry, University of Madras, Guindy Campus, Chennai, 600025 India; 4 CAS in Crystallography & Biophysics, University of Madras, Guindy Campus, Chennai, 600025 India

**Keywords:** Chromen, DFT, Dielectric studies, Molecular docking, Anti-cancer activity

## Abstract

**Background:**

Coumarin (2*H*-chromen-2-one) and its derivatives have a wide range of biological and pharmaceutical activities. They possess antitumor, anti-HIV, anticoagulant, antimicrobial, antioxidant, and anti-inflammatory activities. Synthesis and isolation of coumarins from different species have attracted the attention of medicinal chemists. Herein, we report the synthesis, molecular structure, dielectric, anticancer activity and docking studies with the potential target protein tankyrase.

**Results:**

Molecular structure of (3E)-3-(4-methylbenzylidene)-3,4-dihydro-2*H*-chromen-2-one (MBDC) is derived from quantum chemical calculations and compared with the experimental results. Intramolecular interactions, stabilization energies, and charge delocalization are calculated by NBO analysis. NLO property and dielectric quantities have also been determined. It indicates the formation of a hydrogen bonding between –OH group of alcohol and C=O of coumarin. The relaxation time increases with the increase of bond length confirming the degree of cooperation and depends upon the shape and size of the molecules. The molecule under study has shown good anticancer activity against MCF-7 and HT-29 cell lines. Molecular docking studies indicate that the MBDC binds with protein.

**Conclusions:**

In this study, the compound (3E)-3-(4-methylbenzylidene)-3,4-dihydro-2*H*-chromen-2-one was synthesized and characterized by spectroscopic studies. The computed and experimental results of NMR study are tabulated. The dielectric relaxation studies show the existence of molecular interactions between MBDC and alcohol. Theoretical results of MBDC molecules provide the way to predict various binding sites through molecular modeling and these results also support that the chromen substitution is more active in the entire molecule. Molecular docking study shows that MBDC binds well in the active site of tankyrase and interact with the amino acid residues. These results are compared with the anti cancer drug molecule warfarin derivative. The results suggest that both molecules have comparable interactions and better docking scores. The results of the antiproliferative activity of MBDC and Warfarin derivative against MCF-7 breast cancer and HT-29 colon cancer cell lines at different concentrations exhibited significant cytotoxicity. The estimated half maximal inhibitory concentration (IC 50) value for MBDC and Warfarin derivative was 15.6 and 31.2 μg/ml, respectively. This enhanced cytotoxicity of MBDC in MCF-7 breast cancer and HT-29 colon cancer cell lines may be due to their efficient targeted binding and eventual uptake by the cells. Hence the compound MBDC may be considered as a drug molecule for cancer.

**Graphical abstract:**
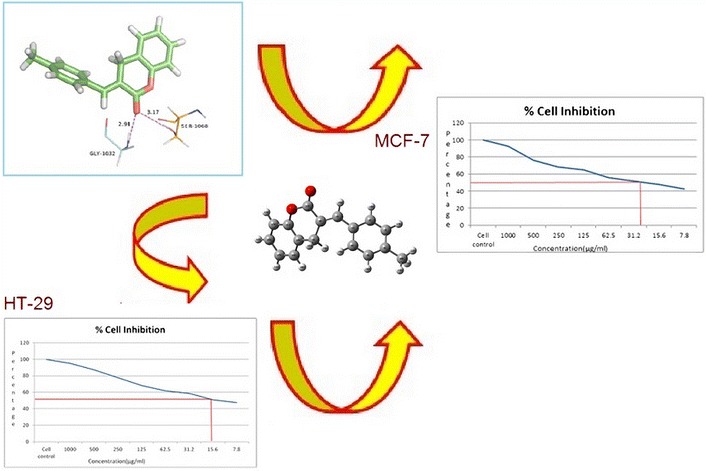
The binding mode of the ligand MBDC at active site of protein and the graphical representation of cell inhibition for MCF-7 and HT-29 cell lines.

## Background

Coumarin (2*H*-chromen-2-one) is one of the important secondary metabolic derivatives which occurs naturally in several plant families. Coumarins are used as a fragrance in food and cosmetic products. Coumarins are widely distributed in the plant kingdom and are present in notable amounts in several species, such as Umbelliferae, Rutaceae and Compositae.

Coumarin and its derivatives have a wide range of biological and pharmaceutical activities. They possess antitumor [1], anti-HIV [2], anticoagulant [3], antimicrobial [4], antioxidant [5] and anti-inflammatory [6] activities. The antitumor activities of coumarin compounds have been extensively examined [7]. Synthesis and isolation of coumarins and its derivatives from different species have attracted the attention of medicinal chemists. The spectroscopic studies led to the beneficial effects on human health and their vibrational characteristics [8, 9].

Herein, we report the synthesis, the computed electronic structure and their properties in comparison with experimental FT-IR, FT Raman, UV and NMR spectra. Further, intra and inter molecular interactions, HOMO–LUMO energies, dipole moment and NLO property have been determined. The dielectric studies confirm the molecular interactions and the strength of hydrogen bonding between the molecule and the solvent ethanol. In addition, anti-cancer activity against MCF-7 and HT-29 cell lines and molecular docking studies have also been performed.

### Experimental

#### Preparation of MBDC

**Scheme 1:**
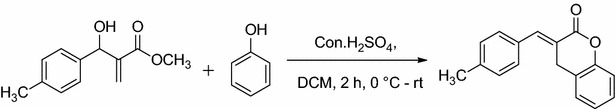
Reaction scheme showing the synthesis of the compound (MBDC)

MBDC was synthesised from the mixture of methyl 2-[hydroxy(4-methylphenyl)methyl]prop-2-enoate (0.206 g, 1 mmol) and phenol (0.094 g, 1 mmol) in CH_2_Cl_2_ solvent and allowed to cool at 0 °C. To this solution, concentrated H_2_SO_4_ (0.098 g, 1 mmol) was added and stirred well at room temperature (Scheme 1). After completion of the reaction as indicated by TLC, the reaction mixture was neutralized with 1 M NaHCO_3_ and then extracted with CH_2_Cl_2_. The combined organic layers were washed with brine (2 × 10 ml) and dried over anhydrous sodium sulfate. The organic layer was evaporated and the residue was purified by column chromatography on silica gel (100–200) mesh, using ethyl acetate and hexane (1:9) as solvents. The pure form of the title compound was obtained as a colorless solid (0.162 g). Yield: 65%, melting point: 132–134 °C.

### Instrumentation

FTIR, FT-Raman, UV–Vis and NMR spectra were recorded using Bruker IFS 66 V spectrometer, FRA 106 Raman module equipped with Nd:YAG laser source, Beckman DU640 UV/Vis spectrophotometer and Bruker Bio Spin NMR spectrometer with CDCl_3_ as solvent, respectively. The dielectric constant (ε′) and dielectric loss (ε″) at microwave frequency were determined by X-Band microwave bench and the dielectric constant (ε_∞_) at optical frequency was determined by Abbe’s refractometer equipped by M/s. Vidyut Yantra, India. The static dielectric constant (ε_0_) was measured by LCR meter supplied by M/s. Wissenschaijftlich Technische, Werkstatter, Germany. Anticancer activity for two cell lines was obtained from National Centre for Cell Sciences, Pune (NCCS).

### Cell line and culture

*MCF*-*7 and HT*-*29* cell lines were obtained from National Centre for Cell Sciences, Pune (NCCS). The cells were maintained in Minimal Essential Medium supplemented with 10% FBS, penicillin (100 U/ml), and streptomycin (100 μg/ml) in a humidified atmosphere of 50 μg/ml CO_2_ at 37 °C.

### Reagents

MEM was purchased from Hi Media Laboratories, Fetal Bovine Serum (FBS) was purchased from Cistron laboratories trypsin, methylthiazolyl diphenyl-tetrazolium bromide (MTT) and dimethyl sulfoxide (DMSO) were purchased from (Sisco Research Laboratory Chemicals, Mumbai). All of other chemicals and reagents were obtained from Sigma Aldrich, Mumbai.

### In vitro assay for anticancer activity (MTT assay)

Cells (1 × 10^5^/well) were plated in 24-well plates and incubated at 37 °C with 5% CO_2_ condition. After the cell reaches the confluence, the various concentrations of the samples were added and incubated for 24 h. After incubation, the sample was removed from the well and washed with phosphate-buffered saline (pH 7.4) or MEM without serum. 100 µl/well (5 mg/ml) of 0.5% 3-(4,5-dimethyl-2-thiazolyl)-2,5-diphenyl-tetrazolium bromide (MTT) was added and incubated for 4 h. After incubation, 1 ml of DMSO was added in all the wells. The absorbance at 570 nm was measured with UV-Spectrophotometer using DMSO as the blank. The %cell viability was calculated using the following formula:

$$\% {\text{cell viability }} = \frac{{\text{ A}}570{\text{ of treated cells }}}{{\text{ A}}570{\text{ of control cells }}} \times 100$$

### Computational methods

Electronic structure and optimized geometrical parameters were calculated by density functional theory (DFT) using Gaussian 09W software package [10] with B3LYP/6-31 + G(d,p) basis set method and Gauss-View molecular visualization program package on a personal computer [11]. Vibrational normal mode wavenumbers of MBDC were derived with IR intensity and Raman intensity. The entire vibrational assignments were executed on the basis of the potential energy distribution (PED) of vibrational modes from VEDA 4 program and calculated with scaled quantum mechanical (SQM) method. The X-ray crystal structure of tankyrase (PDB ID: 4L2K) [12] was obtained from Protein Data Bank (PDB). All docking calculations were performed using induced-fit-docking module of Schrödinger suite [13].

## Results and discussion

### Molecular geometry

**Fig. 1:**
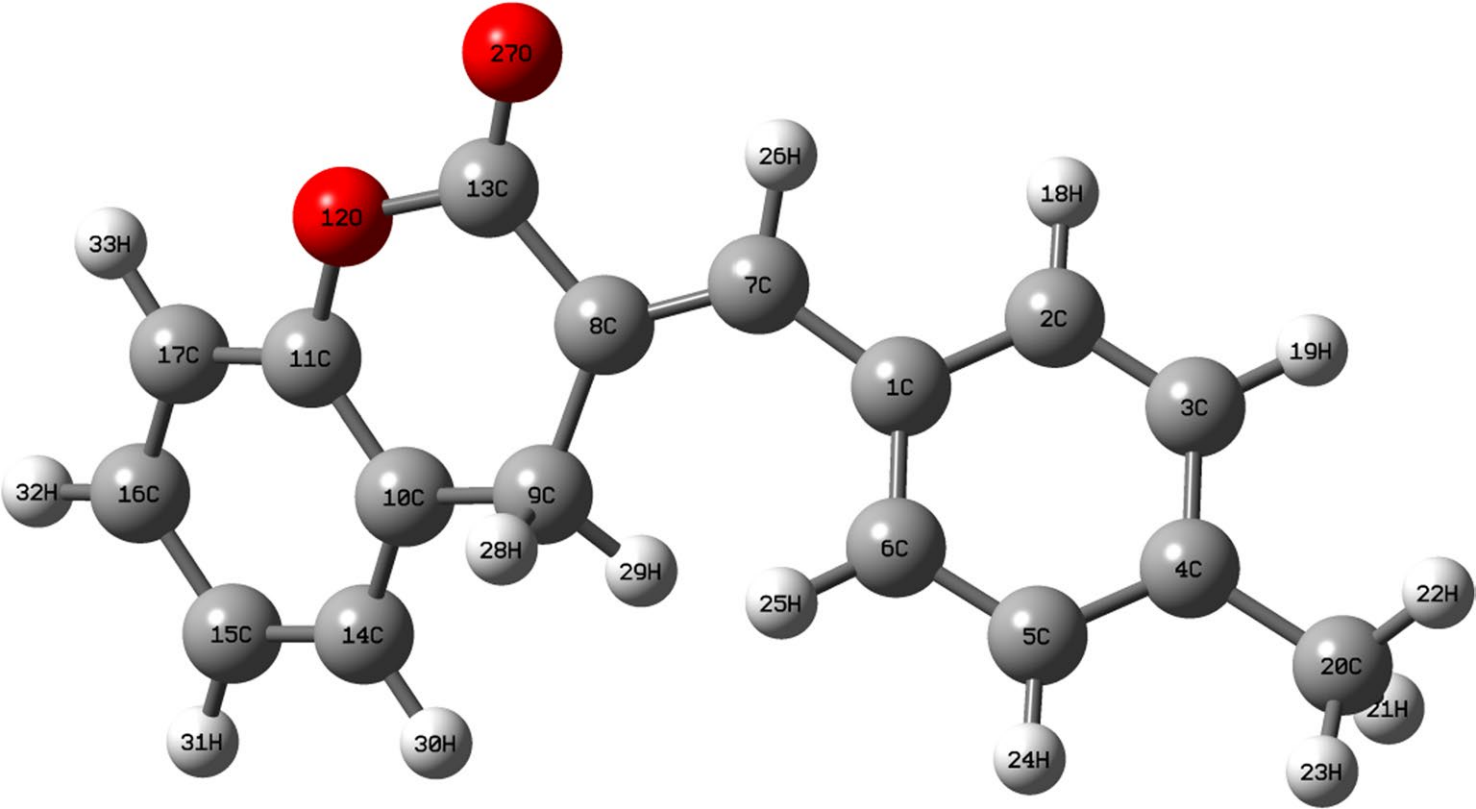
Optimized molecular structure and atomic numbering of MBDC

The optimized molecular structure of MBDC along with the numbering of atoms is shown in Fig. 1. The calculated and experimental bond lengths and bond angles are presented in Table 1. The molecular structure of the compound is obtained from Gaussian 09W and GAUSSVIEW program. The optimized structural parameters (bond lengths and bond angles) calculated by DFT/B3LYP with 6-31 + G(d,p) basis set are compared with experimentally available X-ray data for benzylidene [14] and coumarin [15].

**Table 1:** Optimized geometrical parameters of (3E)-3-(4-methylbenzylidene)-3,4-dihydro-2*H*-chromen-2-one at B3LYP/6-31 + G(d,p) level of theory

Bond length	Value (Å)	Expt.^a^	Bond angle	Value (°)	Expt.^a^
C1–C2	1.411	1.407 (15)	C2–C1–C6	117.36	118.8 (14)
C1–C6	1.408		C6–C1–C7	124.68	124.0 (15)
C1–C7	1.464	1.456 (14)	C1–C2–H31	121.38	120.2 (15)
C2–C3	1.390	1.378 (14)	C3–C2–H18	119.56	119.0 (14)
C2–H18	1.086	0.950 (15)	C2–C3–C4	121.06	121.5 (15)
C3–C4	1.404	1.378 (14)	C3–C4–C5	117.74	117.3 (15)
C3–H19	1.087	0.990 (15)	C3–C4–C20	120.92	120.3 (15)
C4–C5	1.401	1.403 (15)	C5–C6–H25	118.79	119.8 (15)
C4–C20	1.509	1.499 (14)	C1–C7–C8	130.11	131.9 (14)
C5–C6	1.394	1.389 (14)	C8–C7–H26	114.99	
C5–H24	1.087	0.990 (15)	C7–C8–C13	115.44	116.8 (14)
C6–H25	1.083		C7–C8–C9	126.11	125.5 (14)
C7–C8	1.355		C8–C9–C10	112.38	
C7–H26	1.088	0.950 (15)	C8–C9–H28	109.63	
C8–C9	1.511		C8–C9–H29	108.74	
C8–C13	1.491	1.491 (14)	H28–C9–H29	106.06	107.2 (15)
C9–C10	1.509		C9–C10–C11	119.35	
C9–H28	1.102		C9–C10–C14	122.68	
C10–C11	1.394		C8–C13–O27	125.15	
C10–C14	1.400		C10–C14–H30	118.76	
C11–O12	1.387		O12–C11–C17	116.22	116.6 (15)
C11–C17	1.395		C9–C8–C13	118.44	118.96 (14)
O12–C13	1.376		C11–C10–C14	117.93	
C13=O27	1.211	1.261 (15)	C1–C7–H26	114.86	
C14–H30	1.087		C1–C6–C5	120.92	120.7 (14)
C15–C16	1.399		C1–C6–H25	120.23	
C17–H33	1.084		C2–C3–H19	119.40	119.8 (15)
			C10–C11–O12	121.79	120.8 (15)

^a^X-ray data from Refs. [14] and [15]

From the structural data, it is observed that the various C–C bond distances calculated between the rings 1 and 2 and C–H bond lengths are comparable with that of the experimental values of benzylidene and coumarins. The influence of substituent groups on C–C bond distances of ring carbon atoms seems to be negligibly small except that of C3–C4 (1.404 Å) bond length which is slightly longer than the normal value.

The calculated bond lengths of C8–C13 and C4–C20, are 1.491 and 1.509 Å in the present molecule and comparable with the experimental values of 1.491 and 1.499 Å. The experimental value for the bond C13–O7 (1.261 Å) is little longer than the calculated value 1.211 Å. The C–H bond length variations are due to the different substituent’s in the ring and other atoms [16]. The hyper-conjugative interaction effect leads to the deviation of bond angle for C10–C11–O12 (121.79°) from the standard value (120.8°).

### Vibrational spectra

**Fig. 2:**
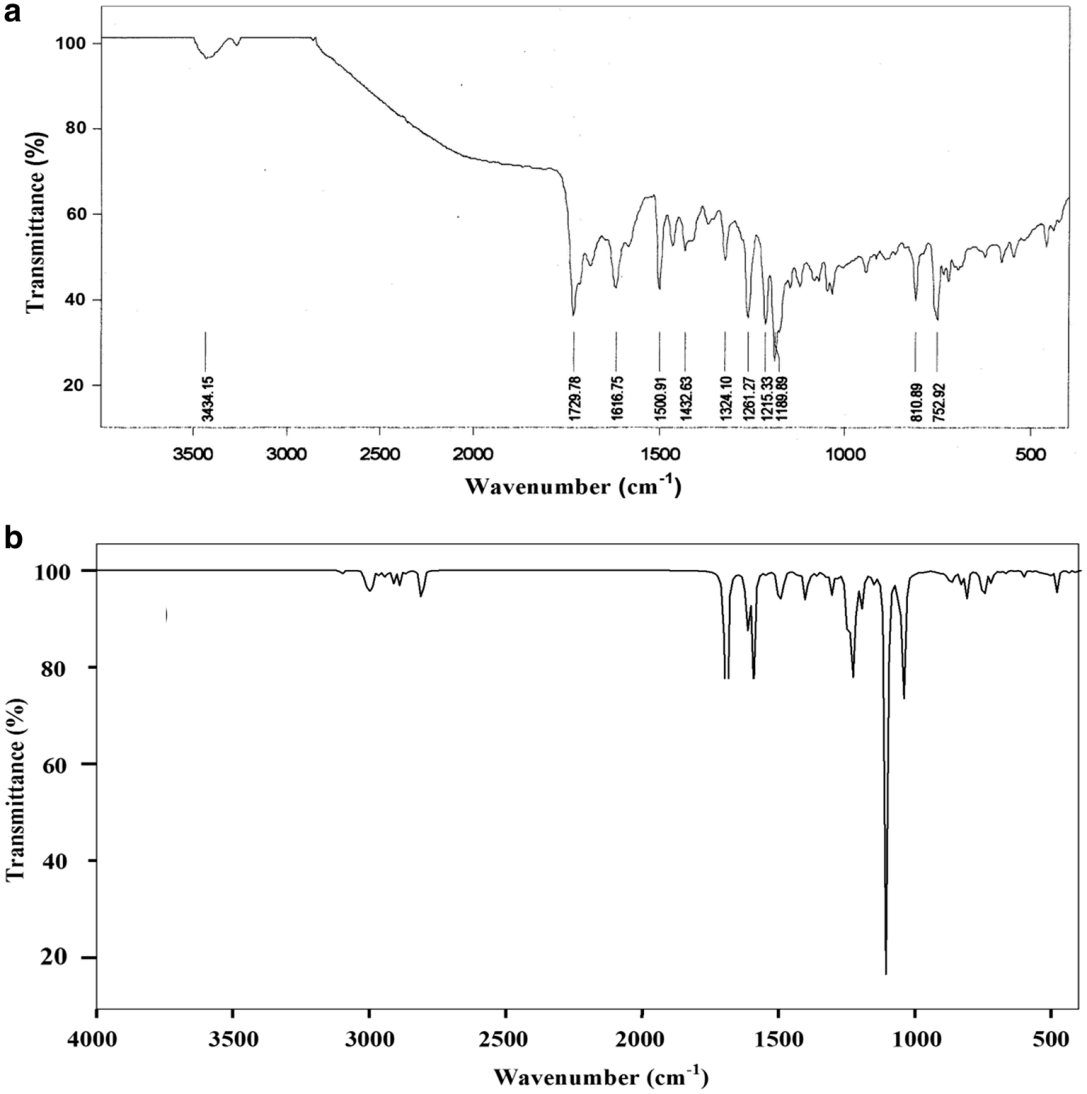
**a** Experimental and **b** predicted FT-IR spectra of MBDC

**Fig. 3:**
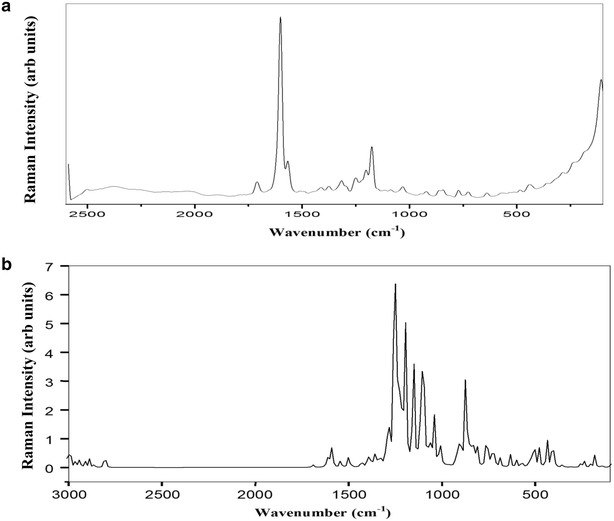
**a** Experimental and **b** predicted FT-Raman spectra of MBDC

The title compound possesses *C*_*s*_ point group symmetry and the available 93 normal modes of vibrations are distributed into two types, namely *A′* (in-plane) and *A″* (out-plane). The irreducible representation for the C_s_ symmetry is given by Г_Vib_ = 63 *A′* + 30 *A″*. All the vibrations are active in both IR and Raman spectra. Vibrational assignments have been carried out from FT-IR (Fig. 2) and FT-Raman (Fig. 3) spectra. The theoretically predicted wavenumbers along with their PED values are presented in Table 2. The fundamental vibrational modes are also characterized by their PED. The calculated wavenumbers are in good agreement with experimental wavenumbers.

**Table 2:** The observed FT-IR, FT-Raman and calculated frequencies (in cm^−1^) using B3LYP/6-31 + G (d,p) along with their relative intensities, probable assignments, reduced mass and force constants of (3E)-3-(4-methylbenzylidene)-3,4-dihydro-2*H*-chromen-2-one

Mode nos	Observed frequencies (cm^−1^)		Calculated frequencies (cm^−1^)		Reduced mass (amu)	Force constant (mdyn/Å)	IR intensity (km/mol)	Raman intensity (Å^4^ amu^−1^)	Vibrational assignments (PED%)
	FTIR	FT Raman	Unscaled	Scaled					
1			23	20	4.139	0.001	0.140	98.862	τ Ring (56), τ CH_3_ (20)
2		30	36	29	1.041	0.001	0.259	2.839	τ Ring (56), τ CH_3_ (20)
3		43	48	42	4.317	0.006	0.138	4.698	τ Ring (55), τ CH_3_ (18)
4		60	61	60	4.037	0.009	0.126	4.758	τ Ring (56), τ CH_3_ (20)
5			81	78	6.433	0.025	1.029	1.382	τ Ring (55), τ CH_3_ (22)
6			101	96	4.785	0.029	0.456	0.906	γ C=O (58), τ CH_3_ (21)
7			156	143	4.419	0.064	1.546	0.321	τ CH_3_ (56)
8			189	181	3.393	0.072	0.402	1.098	τ CH_2_ (56), γ CH_3_ (18)
9		200	225	202	6.604	0.197	2.382	0.235	γ C–CH_3_ (54), γ CH (18), γ CH_3_ (12)
10			252	237	4.366	0.164	1.529	0.314	γ CC (62), γ CH (20), γ CH_2_ (10)
11			274	255	4.050	0.179	1.403	0.314	γ CCC (60), γ CH (22), γ CH_3_ (11)
12			314	286	4.114	0.240	0.632	0.065	γ CCC (59), γ CH (18), γ CH_3_ (10)
13			327	309	5.288	0.335	1.339	0.029	γ CCC (58), γ CH (18), γ CH_3_ (11)
14		350	368	354	3.122	0.249	0.038	0.119	γ CCC (60), γ CH (22), γ CH_3_ (12)
15		400	409	400	3.550	0.350	1.104	0.482	γ CCC (62), γ CH (18), γ CH_3_ (10)
16			421	413	2.977	0.310	1.829	0.326	γ CCC (62), γ CH (20), γ CH_3_ (10)
17			444	437	4.136	0.482	3.120	0.773	γ CCC (62), γ CH (20), γ CH_3_ (11)
18		450	457	453	4.033	0.496	3.817	0.144	γ CCC (63), γ CH (18), γ CH_3_ (12)
19			490	479	5.515	0.783	24.603	0.378	βC=O (58), βCC (22), βCO (10)
20		500	524	506	2.790	0.452	12.486	0.794	γ C–O (64), γ CH_3_ (23), γ CO (10)
21			540	527	5.569	0.786	5.539	0.239	γ CH (58), γ CH_3_ (22), γ CC (10)
22		540	545	540	3.662	0.642	4.599	0.033	γ CH (58), γ CC (21), γ CH_2_ (11)
23		575	582	572	6.588	1.319	2.309	0.138	γ CH (58), γ CH_3_ (20), γ CC (11)
24		600	639	601	6.329	1.526	7.519	0.104	γ CH (56), γ CC (20), γ CH_3_ (10)
25			650	633	6.834	1.703	0.662	0.176	γ CH (58), γ CC (18), γ CH_2_ (11)
26			693	669	5.112	1.447	4.947	0.007	γ CH (56), γ CH_3_ (18), γ CC (12)
27			711	689	3.832	1.142	0.262	0.116	γ CH (56), γ CC (16)
28			727	716	3.876	1.208	9.921	0.085	γ CH (56), γ CC (18)
29		725	737	723	5.549	1.776	11.299	0.128	γ CH (58), γ CC (18)
30			740	735	4.346	1.404	0.599	0.184	βC–CH_3_ (60), βCH (23)
31	750		768	748	1.335	0.465	62.541	0.034	βC–O (62), βCC (22)
32			778	760	4.144	1.481	7.458	0.587	βCC (58), βCH (21), βCH_3_ (10)
33	810		829	811	1.610	0.653	37.872	0.230	βCCC (63), βCH (21), βCH_3_ (12)
34			851	824	1.26	0.540	0.813	0.119	βCCC (63), βCH (18), βCH_3_ (11)
35			858	830	3.739	1.625	14.149	0.099	βCCC (62), βCH_3_ (20), βCH (10)
36			862	838	2.202	0.964	0.532	0.221	βCCC (62), βCH_3_ (21), βCH (12)
37		850	876	850	1.962	0.888	3.587	0.199	βCCC (56), βCH (18), βCH_3_ (10)
38			919	861	6.652	3.314	11.953	0.057	βCCC (58), βCH_3_ (18), βCH (12)
39			947	869	1.572	0.831	5.009	0.061	βCCC (56), βCH (16), βCH_3_ (11)
40		875	954	872	1.399	0.751	11.534	1.087	βCCC (61), βCH (20), βCH_3_ (10)
41			970	889	1.579	0.877	5.474	0.037	βCH (78), ν CC (18)
42		900	981	903	1.476	0.837	5.323	0.410	βCH (76), ν CC (16)
43			984	923	1.377	0.786	2.738	0.150	βCH (78), ν CC (13)
44			988	951	1.282	0.738	0.051	0.002	βCH (66), ν CC (16)
45			1010	968	1.409	0.848	2.809	0.020	βCH (66), ν CC (20)
46		990	1033	992	2.848	1.794	2.530	0.024	βCH (70), ν CC (18)
47			1056	1011	2.122	1.396	3.275	0.289	βCH (76), ν CC (18)
48			1060	1029	1.545	1.024	11.399	0.009	βCH (78), ν CC (17)
49			1088	1042	4.259	2.975	171.99	0.044	βCH (78), ν CC (17)
50		1000	1133	1053	1.775	1.344	19.980	0.028	βCH_2_ipr (67), βCH (20)
51			1148	1061	1.367	1.063	20.088	0.106	γ CH_2_opr (66), βCH (21)
52		1075	1180	1072	1.113	0.914	4.889	0.005	βCH_3_ipr (65), βCC (30)
53		1100	1190	1104	2.389	1.994	564.050	3.029	γ CH_3_opr (71), βCC (23)
54		1150	1215	1153	1.274	1.109	16.185	0.942	ν CO (58), βCH (18), ν CC (11)
55	1189		1218	1190	1.580	1.381	27.443	0.044	ν CO (58), βCH (18), ν CC (12)
56			1227	1197	2.167	1.924	37.004	1.290	ν C=C (82), βCH_3_ (14)
57			1238	1209	2.485	2.247	7.534	0.045	ν CC (71), βCH (16), ν CH_3_ (12)
58			1255	1217	2.115	1.964	33.951	0.281	ν C–CH_3_ (50), βCH (20), βCO (12)
59	1215		1258	1231	3.099	2.893	219.799	0.644	βCH_2_sb (66), βCC (22), βCH (11)
60			1288	1243	1.825	1.785	19.982	0.588	βCH_2_asb (70), βCC (20), βCH (10)
61		1250	1340	1250	5.462	5.782	49.937	0.759	βCH_3_sb (71), βCC (23), βCH (11)
62	1261		1342	1260	1.625	1.727	2.543	0.527	βCH_3_asb (66), βCH (17), ν CC (10)
63			1349	1287	2.373	2.544	13.033	0.436	βCH_3_asb (60), βCH (18), ν CC (10)
64			1369	1306	2.450	2.709	31.517	0.047	ν CC (68), βCH (18)
65			1407	1330	1.776	2.074	9.480	0.143	ν CC (66), βCH (19)
66			1420	1343	1.248	1.483	0.324	0.393	ν CC (66), βCH (18)
67			1440	1362	2.310	2.850	7.463	0.084	ν CC (68), βCH (19)
68			1476	1387	1.277	1.449	12.963	0.069	ν CC (68), βCH (19)
69			1491	1395	1.072	1.450	11.786	0.102	ν CC (70), βCH (18)
70			1492	1404	2.295	3.013	30.676	0.013	ν CC (70), βCH (17)
71	1432		1496	1430	1.114	1.469	9.704	0.119	ν CC (68), βCH (17)
72			1529	1487	2.593	3.574	57.049	0.019	ν CC (66), βCH (18)
73	1500		1548	1502	2.482	3.505	23.043	0.262	ν CC (65), βCH (18)
74		1540	1603	1543	5.415	8.200	5.106	0.867	ν CC (66), βCH (19)
75			1636	1587	6.310	9.958	21.097	0.660	ν CC (65), βCH (18)
76			1654	1592	6.049	9.754	145.323	3.229	ν CC (66), βCH (18)
77			1659	1604	6.840	11.109	9.718	0.093	ν CC (68), βCH (18)
78		1600	1668	1615	7.222	11.846	91.204	0.131	ν CC (70), βCH (16)
79	1616	1690	1793	1692	12.541	23.775	370.738	0.460	ν C=O (72), ν CC (14)
80			2980	2801	1.072	5.615	14.012	0.299	ν ssCH_2_ (80)
81		2800	3034	2809	1.039	5.641	33.955	0.722	ν assCH_2_ (82)
82			3080	2863	1.088	6.085	4.273	0.081	ν ssCH_3_ (72), ν CH (23)
83			3092	2889	1.097	6.182	17.402	0.180	ν assCH_3_ (80), ν CH (16)
84			3122	2911	1.102	6.330	15.019	0.127	ν assCH_3_ (88), ν CH (11)
85			3172	2936	1.088	6.451	3.815	0.088	ν CH (96)
86			3175	2945	1.088	6.464	5.999	0.065	ν CH (96)
87			3177	2962	1.088	6.464	7012	0.109	ν CH (96)
88			3179	2989	1.089	6.488	17.412	0.127	ν CH (98)
89			3192	2993	1.089	6.536	7.580	0.129	ν CH (98)
90			3193	2999	1.094	6.574	14.859	0.219	ν CH (96)
91			3206	3007	1.094	6.629	18.471	0.243	ν CH (98)
92		3020	3218	3018	1.096	6.687	5.949	0.335	ν CH (98)
93		3100	3225	3101	1.091	6.690	6.782	0.076	ν CH (98)

ν, stretching; β, in plane bending; γ, out of plane bending; ω, wagging; τ, torsion; ρ, rocking; δ, scissoring; ss, symmetric stretching; ass, antisymmetric stretching; sb, symmetric bending; asb, antisymmetric bending; ipr, in-plane-rocking; opr, out-of-plane rocking

### Carbon–hydrogen vibrations

The C–H stretching vibrations are expected to appear at 3100−2900 cm^−1^ [17] with multiple weak bands. The four hydrogen atoms left around each benzene ring give rise to a couple of C–H stretching, C–H in-plane bending and C–H out-of-plane bending vibrations. In MBDC, the calculated wavenumbers at 2936, 2945, 2962, 2989, 2993, 2999, 3007, 3018 and 3101 cm^−1^ are assigned to C–H stretching modes which show good agreement with the literature values [18]. The C–H in-plane bending vibrations occur in the region of 1390–990 cm^−1^. The vibrational assignments at 900, 990 and 1000 cm^−1^ (Fig. 3) occur due to the effect of C–H in-plane bending vibrations. The calculated wavenumbers at 889, 903, 923, 951, 968, 992, 1011, 1029 and 1042 cm^−1^ are due to C–H in-plane bending vibrations which show good agreement with recorded spectral values.

The out-of-plane bending of ring C–H bonds occur below 900 cm^−1^ [19]. In MBDC, the C–H out-of-plane bending vibrations are observed at 540, 575, 600 and 725 cm^−1^ which are compared with the computed values at 527, 540, 572, 601, 633, 669, 689, 716 and 723 cm^−1^.

### Carbon–carbon vibrations

The ring C=C and C–C stretching vibrations, known as semicircle stretching modes, usually occur in the region of 1625–1400 cm^−1^ [20]. Generally, these bands are of variable intensity and observed at 1625–1590 cm^−1^, 1590–1575 cm^−1^, 1540–1470 cm^−1^, 1465–1430 cm^−1^ and 1380–1280 cm^−1^ [21]. In MBDC, the aromatic C–C stretching vibrations are observed at 1209 cm^−1^ (Fig. 2). The C–C stretching vibrations are assigned at 1432 and 1500 cm^−1^ in FT-IR and at 1540 and 1600 cm^−1^ in FT-Raman spectrum. These values perfectly match with the calculated wavenumbers, 1306–1615 cm^−1^ (mode no. 64–78). The C–C–C in-plane bending vibrations are observed at 810 cm^−1^ in FT-IR spectrum and at 850 and 875 cm^−1^ in FT-Raman spectrum. The calculated values are 811–872 cm^−1^ (mode no: 33–40). The C–C–C out-of-plane bending vibrations appeared at 350 and 400 cm^−1^ in FT-Raman spectrum and the corresponding calculated wavenumbers at 255–453 cm^−1^ (mode no: 11–18) show good agreement with the literature values [16]. These observed wavenumbers show that the substitutions in the benzene ring affect the ring modes of vibrations to a certain extent.

### C–O vibrations

The C–O stretching vibrations are observed at 1300–1200 cm^−1^ [22]. In the present molecule, the C–O stretching is observed at 1189 cm^−1^ in FT-IR spectrum and the calculated vibration is at 1153 and 1190 cm^−1^. The C–O in-plane bending vibration is observed at 750 cm^−1^ in FT-IR matches with the theoretical value of 748 cm^−1^. In this molecule, the peak observed at 500 cm^−1^ in FT-Raman and 506 cm^−1^ in FT-IR are attributed to C–O out-of-plane bending vibrations. The C=O stretching vibration is generally observed at 1800–1600 cm^−1^ [23]. In MBDC, the C=O stretching is observed at 1616 cm^−1^ in FT-IR and at 1690 cm^−1^ in FT-Raman spectrum. This peak matches with the calculated value (1692 cm^−1^).

### CH_2_ vibrations

The asymmetric CH_2_ stretching vibrations are generally observed between 3000 and 2800 cm^−1^, while the symmetric stretch appears between 2900 and 2800 cm^−1^ [24]. In MBDC, the CH_2_ asymmetric and symmetric stretching vibrations are calculated at 2809 and 2801 cm^−1^ respectively. The asymmetric bending is calculated at 1243 cm^−1^. In FT-IR spectrum the symmetric bending vibration is observed at 1215 cm^−1^ and calculated at 1231 cm^−1^. The in-plane CH_2_ bending vibration is observed at 1000 cm^−1^ in FT-Raman spectrum and the calculated vibration is at 1053 cm^−1^. The out-of-plane CH_2_ bending vibration is calculated at 1061 cm^−1^. The above results suggest that the observed frequencies are in good agreement with calculated in-plane and out-of-plane modes.

### CH_3_ vibrations

There are nine fundamental modes associated with each CH_3_ group. In aromatic compounds, the CH_3_ asymmetric and symmetric stretching vibrations are expected in the range of 2925–3000 cm^−1^ and 2905–2940 cm^−1^, respectively [25]. In CH_3_ antisymmetric stretching mode, two C–H bonds are expanding while the third one is contracting. In symmetric stretching, all the three C–H bonds are expanding and contracting in-phase. In MBDC, the assigned vibrations at 2911, 2889 and 2863 cm^−1^ represent asymmetric and symmetric CH_3_ stretching vibrations [26]. The CH_3_ symmetric bending vibrations are observed at 1250 cm^−1^ in FT-Raman spectrum and calculated at 1250 cm^−1^ which are in good agreement with experimental and theoretical vibrations. The CH_3_ asymmetric bending vibrations are observed at 1261 cm^−1^ and calculated at 1260 and 1287 cm^−1^ match with the experimental values. The in-plane CH_3_ bending vibration is assigned at 1075 cm^−1^ in FT-Raman and calculated at 1072 cm^−1^ in B3LYP and out-of-plane CH_3_ bending vibration is observed at 1100 cm^−1^ in FT-Raman and calculated at 1104 cm^−1^. Predicted wavenumbers derived from B3LYP/6-31 + G(d,p) method synchronise well with those of the experimental observations.

### HOMO–LUMO analysis

**Fig. 4:**
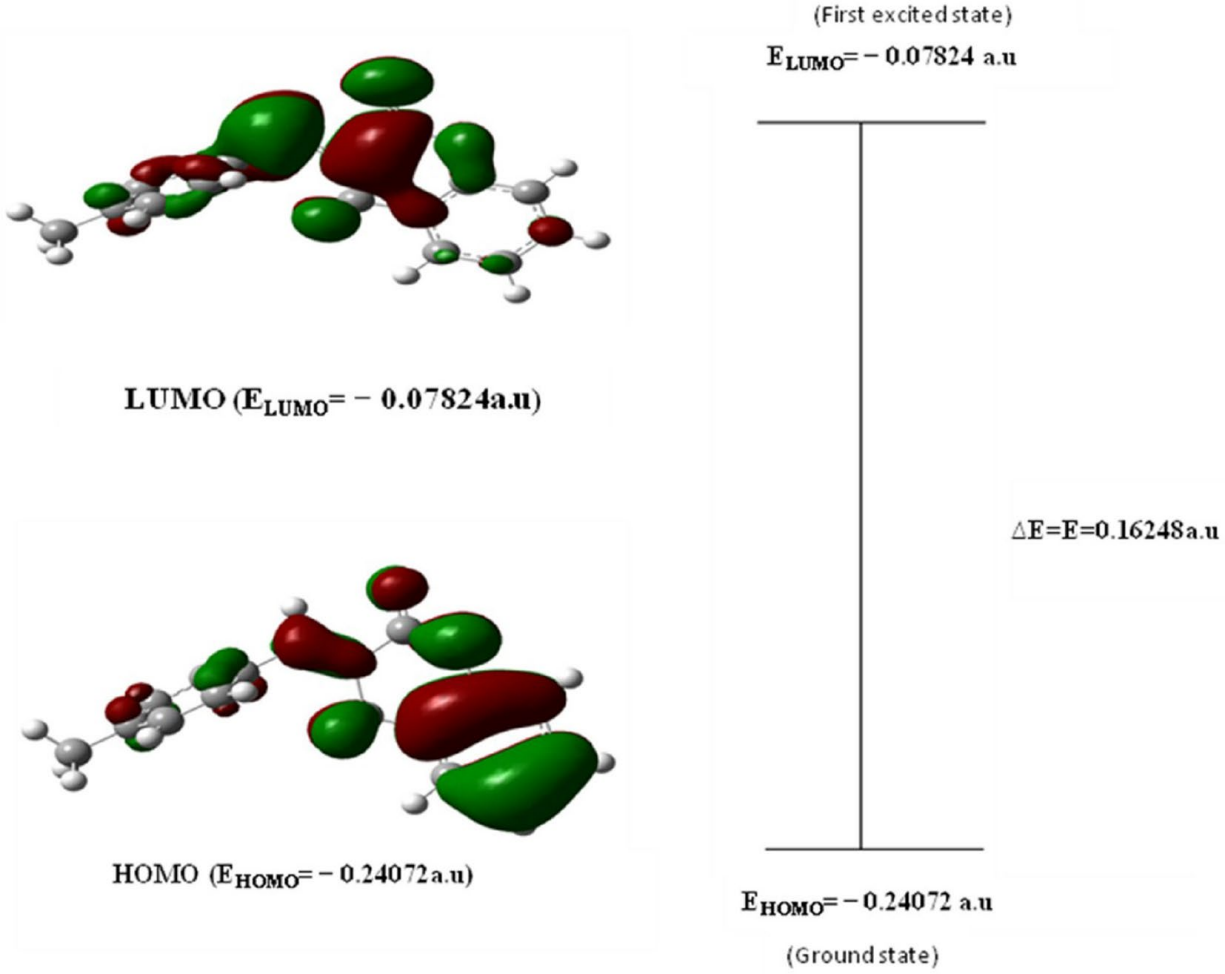
The calculated frontiers energies of MBDC

The most important orbitals in the molecule is the frontier molecular orbitals, called highest occupied molecular orbital (HOMO) and lowest unoccupied molecular orbital (LUMO). These orbitals determine the way the molecule interacts with other species. The HOMO–LUMO energy gap of MBDC is shown in Fig. 4. The HOMO (−51.0539 kcal/mol) is located over the coumarin group and LUMO (−49.0962 kcal/mol) is located over the ring; the HOMO→LUMO transition implies the electron density transfer to ring benzylidene. The calculated self-consistent field (SCF) energy of MBDC is −506,239.7545 kcal/mol. The frontier orbital gap is found to be E = −101.9576 kcal/mol and this negative energy gap confirms the intramolecular charge transfer. This proves the non-linear optical (NLO) activity of the material [27]. A molecule with a small frontier molecular orbital is more polarizable and generally associated with high chemical reactivity, low kinetic stability termed as soft molecule [28]. The low value of frontier molecular orbital in MBDC makes it more reactive and less stable.

### NBO analysis

Natural bond orbital (NBO) of the molecule explains the molecular wave function in terms of Lewis structures, charge, bond order, bond type, hybridization, resonance, donor–acceptor interactions, etc. NBO analysis has been performed on MBDC to elucidate the intramolecular, rehybridization and also the interaction which will weaken the bond associated with the anti-bonding orbital. Conversely, an interaction with a bonding pair will strengthen the bond.

The corresponding results are presented in Tables 3 and 4. The intramolecular interaction between lone pair of O27 with antibonding C13–O12 results in a stabilized energy of 35.64 kcal/mol. The most important interaction in MBDC is between the LP(2)O12 and the antibonding C13–O27. This results in a stabilization energy 41.74 kcal/mol and denotes larger delocalization. The valence hybrid analysis of NBO shows that the region of electron density distribution mainly influences the polarity of the compound. The maximum electron density on the oxygen atom is responsible for the polarity of the molecule. The p-character of oxygen lone pair orbital LP(2) O27 and LP(2) O12 are 99.66 and 99.88, respectively. Thus, a very close pure p-type lone pair orbital participates in the electron donation in the compound.

**Table 3:** Second-order perturbation energy [E(2), kcal/mol] between donor and acceptor orbitals of MBDC calculated at B3LYP/6-31 + G(d,p) level of DFT theory

Donor (i)	Acceptor (j)	E(2)	ED (i) (e)	ED (j)(e)	E(j) − E(i) (a.u.)	F(i,j) (a.u.)
LP(1)O_27_	σ*C_8_–C_13_	3.01	1.97789	0.07355	1.11	0.052
LP(1)O_27_	σ*C_13_–O_12_	0.08	1.97789	0.10629	1.03	0.026
LP(2)O_27_	π*C_8_–C_13_	18.58	1.83804	0.07355	0.67	0.102
LP(2)O_27_	π*C_13_–O_12_	35.64	1.83804	0.10629	0.60	0.132
LP(2)O_27_	π*C_7_–H_26_	0.70	1.83804	0.01944	0.73	0.021
LP(1)O_12_	σ*C_8_–C_13_	6.30	1.95794	0.07355	0.96	0.070
LP(1)O_12_	σ*C_10_–C_11_	6.54	1.95794	0.03331	1.11	0.076
LP(1)O_12_	σ*C_11_–C_17_	0.77	1.95794	0.02024	1.10	0.026
LP(1)O_12_	σ*C_13_–O_27_	2.06	1.95794	0.01348	1.16	0.044
LP(2)O_12_	σ*C_10_–C_11_	25.17	1.95794	0.38783	0.36	0.088
LP(2)O_12_	σ*C_13_–O_27_	41.74	1.76210	0.24560	0.34	0.106
σC_8_–C_9_	σ*C_8_–C_7_	3.21	1.9767	0.01864	1.29	0.057
σC_8_–C_13_	σ*C_7_–C_1_	4.13	1.97727	0.02282	1.14	0.061
πC_9_–H_28_	π*C_8_–C_7_	3.36	1.96228	0.06368	0.55	0.038
πC_9_–H_29_	π*C_10_–C_11_	3.31	1.96216	0.38783	0.53	0.041
σC_10_–C_14_	σ*C_11_–O_12_	4.82	1.97139	0.03516	1.03	0.063
σC_11_–C_17_	σ*C_10_–C_11_	4.15	1.97581	0.03331	1.28	0.065
σH_30_–C_14_	σ*C_10_–C_11_	4.18	1.98112	0.03331	1.10	0.061
σC_17_–C_16_	σ*C_11_–O_12_	4.34	1.97651	0.03516	1.03	0.060
σC_17_–H_33_	σ*C_10_–C_11_	4.56	1.97906	0.03331	1.09	0.063
σC_7_–H_26_	σ*C_8_–C_9_	7.24	1.96715	0.02414	0.94	0.074
σC_2_–H_18_	σ*C_1_–C_6_	4.35	1.98162	0.02521	1.08	0.061
σC_6_–H_25_	σ*C_1_–C_2_	4.31	1.98170	0.02470	1.09	0.061
σC_5_–H_24_	σ*C_6_–C_4_	4.24	1.98119	0.02266	1.00	0.029
πC_20_–H_21_	π*C_5_–C_4_	4.04	1.98750	0.34063	0.53	0.045

**Table 4:** NBO results showing the formation of Lewis and non Lewis orbitals of MBDC molecule by B3LYP/6-31G + (d,p) method

Bond (A–B)	ED/energy (a.u.)	ED_A_ %	ED_B_ %	NBO	s %	p %
σ C8–C9	1.97667	50.31	49.69	0.7093 (sp^2.03^) 0.7049 (sp^2.71^)	32.95 26.97	67.02 72.98
	−0.65200					
σ C8–C13	1.97727	51.86	48.14	0.7201 (sp^2.48^) 0.6938 (sp^1.52^)	28.69 39.66	71.27 60.28
	−0.68595					
σ C9–H28	1.96228	63.78	36.22	0.7986 (sp^3.34^) 0.6019 (sp^0.00^)	23.04 99.95	76.91 00.05
	−0.51190					
σ C10–C14	1.97139	51.60	48.40	0.7184 (sp^1.82^) 0.6957 (sp^1.91^)	35.47 34.37	64.50 65.59
	−0.70409					
σ C11–C17	1.97581	51.16	48.84	0.7153 (sp^1.62^) 0.6989 (sp^2.00^)	38.17 33.31	61.80 66.64
	−0.71570					
σ H30–C14	1.98112	37.66	62.34	0.6137 (sp^0.00^) 0.7896 (sp^2.37^)	99.95 29.65	00.05 70.31
	−0.53074					
σ C17–C16	1.97651	50.46	49.54	0.7103 (sp^1.79^) 0.7039 (sp^1.88^)	35.85 34.75	64.11 65.20
	−0.25929					
σ C17–H33	1.97906	63.18	36.782	0.7948 (sp^2.24^) 0.6068 (sp^0.00^)	30.81 99.95	69.15 00.04
	−0.52986					
σ C7–H26	1.96715	63.87	36.13	0.7992 (sp^2.36^) 0.6011 (sp^0.00^)	29.74 99.95	70.22 00.05
	−0.52611					
σ C2–H18	1.98162	62.58	37.42	0.7911 (sp^2.34^) 0.6117 (sp^0.00^)	29.94 99.95	70.02 00.05
	−0.52927					
σ C6–H25	1.98170	62.53	37.47	0.7908 (sp^2.34^) 0.6121 (sp^0.00^)	29.93 99.95	70.03 00.05
	−0.53031					
σ C5–H24	1.98119	62.30	37.70	0.7893 (sp^2.37^) 0.6140 (sp^0.00^)	29.62 99.95	70.34 00.05
	−0.52761					
σ C20–H21	1.98750	62.42	37.58	0.7901 (sp^3.12^) 0.6130 (sp^0.00^)	24.25 99.95	75.70 00.05
	−0.51049					
LP(1) O27	1.97789			sp^0.70^	58.63	41.30
	−0.69724					
LP(2) O27	1.83804			sp^99.99^	00.05	99.66
	−0.26311					
LP(1) O12	1.95794			sp^1.89^	34.56	65.38
	−0.54749					
LP(2) O12	1.76210			sp^1.00^	00.00	99.88
	−0.33734					

### Mulliken charges

The Mulliken atomic charges of MBDC were calculated by B3LYP/6–31 + G (d,p) level theory (Table 5). It is important to mention that the atoms C1, C2, C4, C7, C10, H18, H19, O27 of MBDC exhibit positive charges, whereas the atoms C3, C5, C6, C11, O12 exhibit negative charges. The maximum negative and positive charge values are −0.95788 for C11 and 0.90500 for C10 in the molecule, respectively.

**Table 5:** The charge distribution calculated by the Mulliken method

Atoms	Mulliken charge	NBO
C_1_	0.35122	−0.09783
C_2_	0.07866	−0.22079
C_3_	−0.25976	−0.23196
C_4_	0.28427	−0.03843
C_5_	−0.54829	−0.23334
C_6_	−0.26856	−0.22441
C_7_	0.10817	−0.12331
C_8_	0.48781	−0.15456
C_9_	−0.49756	−0.50908
C_10_	0.90500	−0.08766
C_11_	−0.95788	0.29617
O_12_	−0.39388	−0.51439
C_13_	0.33449	0.80701
C_14_	−0.31967	−0.21966
C_15_	0.13614	−0.25219
C_16_	−0.08232	−0.23483
C_17_	−0.15764	−0.26075
H_18_	0.13200	0.24986
H_19_	0.12586	0.24422
C_20_	−0.60604	−0.70947
H_21_	0.17095	0.24897
H_22_	0.16101	0.24929
H_23_	0.15358	0.25629
H_24_	0.12235	0.24404
H_25_	0.12453	0.24877
H_26_	0.15765	0.27521
O_27_	−0.44633	−0.56839
H_28_	0.18552	0.27671
H_29_	0.16406	0.27813
H_30_	0.12443	0.24480
H_31_	0.12660	0.24891
H_32_	0.13021	0.25025
H_33_	0.14289	0.26243

### UV–Visible analysis

**Fig. 5:**
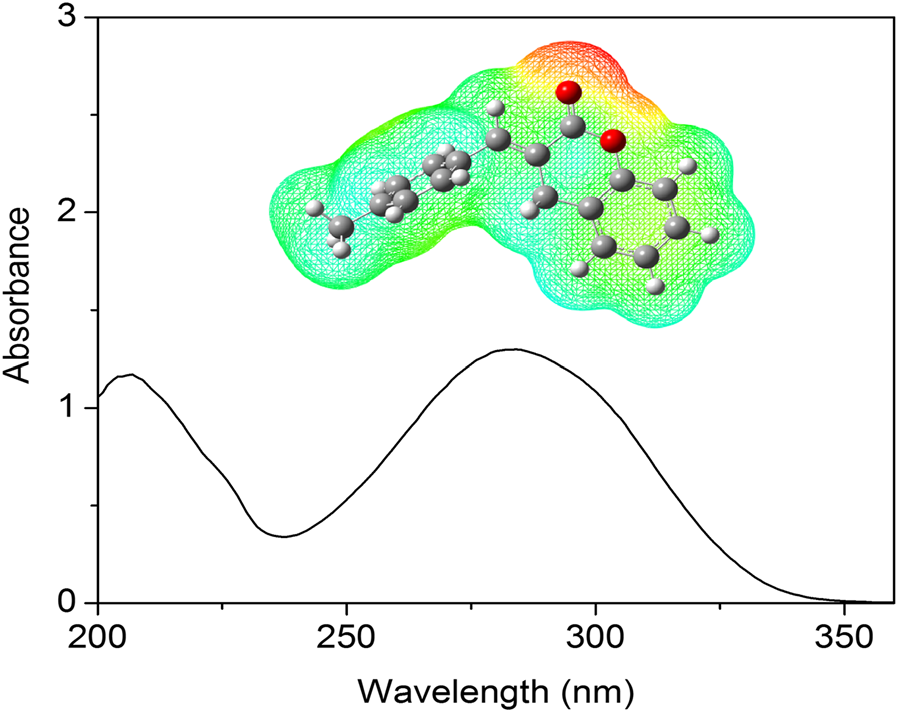
Experimental UV spectrum of MBDC. *Inset figure* predicated MEP map of MBDC

Theoretical UV–Visible spectrum (Table 6) of MBDC was derived by employing polarizable continuum model (PCM) and TD-DFT method with B3LYP/6-31 + G(d,p) basis set and compared with experimentally obtained UV–Visible spectrum (Fig. 5). The spectrum shows the peaks at 215 and 283 nm whereas the calculated absorption maxima values are noted at 223, 265 and 296 nm in the solvent of ethanol. These bands correspond to one electron excitation from HOMO–LUMO. The band at 223 and 265 nm are assigned to the dipole-allowed σ → σ* and π → π* transitions, respectively. The strong transitions are observed at 2.414 eV (215 nm) with *f* = 0.0036 and at 2.268 eV (283 nm) with *f* = 0.002.

**Table 6:** UV-Vis excitation energy and electronic absorption spectra of MBDC using TD-B3LYP/631G + (d,p) method

Exp. (nm)	Wavelength (nm)	Energy (eV)	Oscillator strength (f)	Assignments
283	296	2.2007	0.0134	π → π*
283	265	2.2684	0.002	π → π*
215	223	2.4147	0.0036	σ − σ*

### Molecular electrostatic potential

Molecular electrostatic potential at the surface are represented by different colours (inset in Fig. 5). Red colour indicates electronegative character responsible for electrophilic attack, blue colour indicates positive region representing nucleophilic attack and green colour represents the zero potential. The electrostatic potential increases in the order red < orange < yellow < green < blue [29]. The mapped electrostatic potential surface of the molecule shows that atoms O27 and O12 of chromen possess negative potential and all H atoms have positive potential. The same regions are identified in the Mulliken charges also.

### Hyper polarizability

On the basis of the finite-field approach, using B3LYP/6–31 + G (d,p) basis set, the first hyperpolarizability (*β*), dipole moment (*μ*) and polarizability (*α*) for MBDC are calculated and compared with urea (Table 7) [30]. The dipole moment of MBDC is 1.6941 times greater than the magnitude of urea (*μ*_tot_ of urea is 3.2705 D) and the first hyperpolarizability is 1.51 times greater than the magnitude of urea (*β*_*tot*_ of urea is 3.7472 × 10^−31^ esu). Urea is the standard NLO crystal reported earlier [31] so that a direct comparison was made.

**Table 7:** The calculated electric dipole moment (μ_tot_ D) the average polarizability (α_tot_ × 10^−24^ esu) and the first hyperpolarizability (β_tot_ × 10^−31^ esu)

Parameters	Values
μ_x_	2.9237
μ_y_	−4.6995
μ_z_	−0.2541
μ_tot_ (D)	5.5406
α_xx_	−93.6767
α_xy_	6.1433
α_yy_	−119.8535
α_xz_	−0.1725
α_yz_	−4.4825
α_zz_	−111.9369
α_tot_ (esu)	2.32632 × 10^**−**24^
β_xxx_	23.1945
β_xxy_	−28.7842
β_xyy_	20.1351
β_yyy_	−51.2342
β_xxz_	−32.9779
β_xyz_	−12.6553
β_yyz_	−7.0618
β_xzz_	5.9903
β_yzz_	8.6308
β_zzz_	6.4779
β_tot_ (esu)	5.6583 × 10^−31^

### Dielectric studies

The experimental data of ε_0_, ε′, ε_∞_ and τ of MBDC in ethanol at various concentrations are presented in Table 8. The static and microwave dielectric constants decrease with increasing concentration of the compound. This shows a weak interaction exists between the molecule and the solvent at low frequencies. Optical dielectric constant increases with increasing solute concentration which leading to a strong interaction between MBDC and ethanol at high frequency. It indicates the formation of a hydrogen bonding between –OH group of alcohol and C=O of coumarin. The relaxation time increases with the increase of bond length confirming the degree of cooperation, shape and size of the molecule [32].

**Table 8:** Values of dielectric constant (ε_0_, ε′, ε_∞_) and relaxation time τ(ps) of MBDC in ethanol at 303 K

System	Mole conc.	Static dielectric constant (ε_0_)	Microwave dielectric constant (ε′)	Optical dielectric constant (ε_∞_)	Relaxation time τ (ps)
Ethanol + MBDC	0.025	24.10	22.45	1.848	125.45
	0.040	21.14	20.33	1.945	132.61
	0.055	19.36	18.39	2.570	148.44
	0.070	15.89	16.59	2.832	153.89

### NMR study

**Fig. 6:**
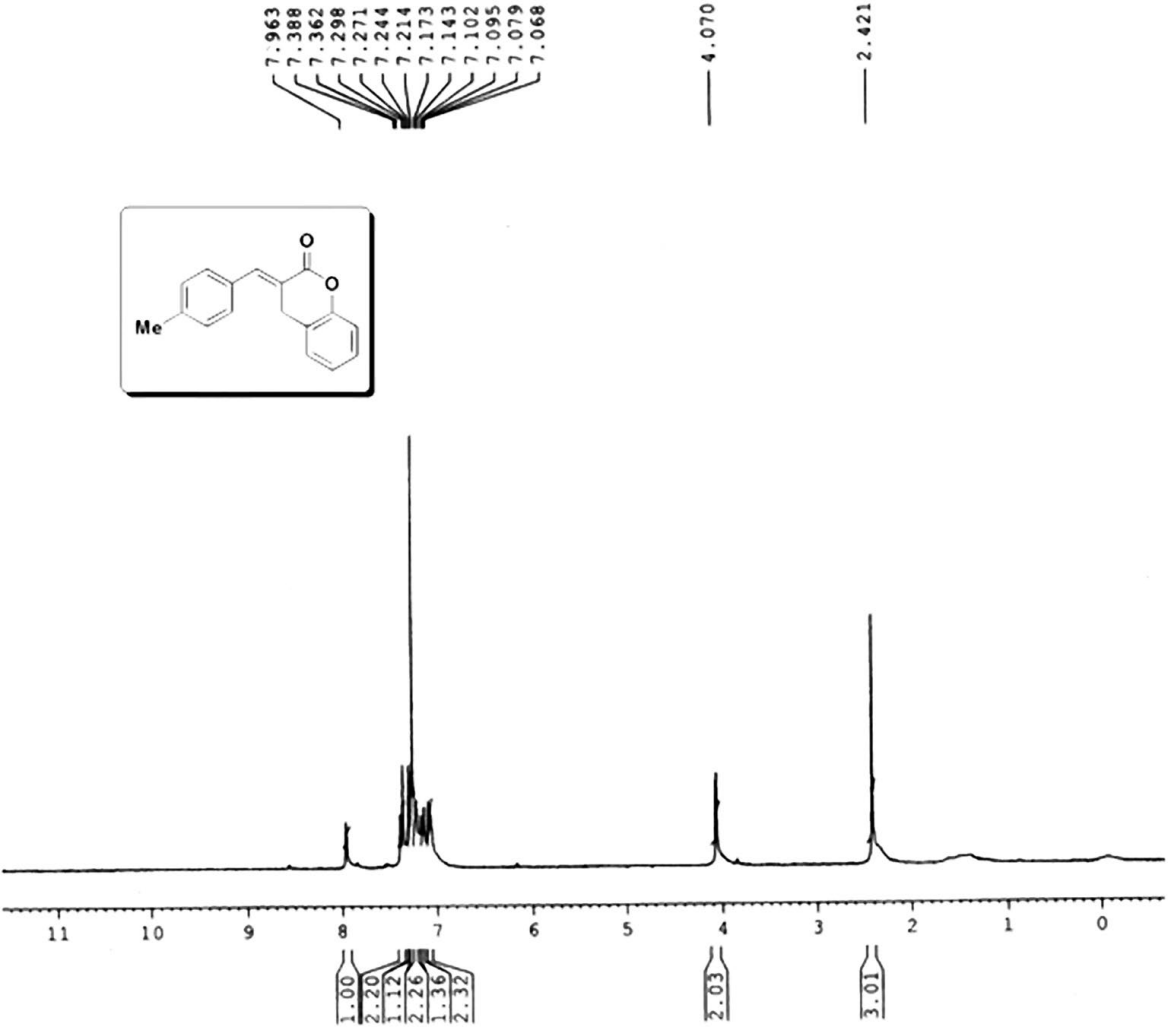
Experimental ^1^H NMR spectrum of MBDC

The characterization of MBDC was further enhanced by the study of ^1^H NMR method. The computed ^13^C NMR and ^1^H NMR chemical shifts and experimental ^1^H NMR are compiled in Table 9. The experimental ^1^H NMR spectrum in CDCl_3_ solution is shown in Fig. 6. The relevant difference of ^1^H NMR chemical shifts calculated by GIAO/B3LYP method is: 0.06(H31), 0.17(H26) and 0.19(H24). The maximum deviation from experimental value is responded to be 0.19 ppm for H24 atom [33]. Overall the calculated values agree with the experimental chemical shift values and the slight deviations may be due to the influence of proton exchange, hydrogen bond and solvent effect in complex real systems. The results of ^13^C NMR chemical shift of the MBDC compound is reliable for the interpretation of spectroscopic parameters. The C1 and C2 atoms of the compound are attached with the electron releasing group and hence they are more electron donating than C15. This causes more shielding at C1 and C2 positions and hence the chemical shift values are lesser.

**Table 9:** Experimental (in CDCl_3_), predicted (δ_pred_) ^13^C and ^1^H chemical shifts (ppm) and calculated GIAO/B3LYP/6-31 + G(d,p) isotropic magnetic shielding tensors (σ_calc_) for (3E)-3-(4-methylbenzylidene)-3,4-dihydro-2*H*-chromen-2-one

^1^H	δ_exp_ (CDCl_3_)	CDCl_3_		Gas phase		^13^C	CDCl_3_		Gas phase	
		δ_pred_	σ_calc_	δ_pred_	σ_calc_		δ_pred_	σ_calc_	δ_pred_	σ_calc_
H18	7.36	7.42	23.9144	7.20	24.1513	C1	115.85	62.9668	116.66	62.1766
H19	7.36	7.46	23.8777	7.22	24.1263	C2	117.49	61.3681	117.18	61.6766
H21	2.42	2.66	28.8984	2.63	28.9317	C3	111.81	66.8779	111.47	67.2105
H22	2.42	2.39	29.1857	2.34	29.2393	C4	127.41	51.7495	125.56	53.5485
H23	2.42	2.21	29.3704	2.14	29.4509	C5	111.58	67.1015	111.27	67.4047
H24	7.21	7.40	23.9349	7.15	24.2029	C6	112.70	66.0193	112.14	66.5622
H25	7.39	7.41	23.9272	7.24	24.1070	C7	129.24	49.9746	127.65	51.5188
H26	7.96	8.13	23.1789	8.01	23.3020	C8	106.14	72.3815	106.55	71.98
H28	4.07	4.08	27.4169	3.92	27.5850	C9	15.45	160.332	16.03	159.7719
H29	4.07	4.02	27.4732	3.92	27.5830	C10	106.20	72.3198	104.77	73.708
H30	7.24	7.25	24.0981	6.95	24.4081	C11	134.84	44.5441	135.63	43.7844
H32	7.28	7.33	24.0134	7.10	24.2574	C13	149.18	30.6419	146.48	33.261
H33	7.10	7.10	24.2534	6.93	24.4260	C14	110.11	68.5299	109.42	69.2007
						C15	107.00	71.5493	105.72	72.7857
						C16	109.94	68.6951	109.65	68.9804
						C17	99.92	78.414	100.35	77.9959

### Molecular docking studies

**Fig. 7:**
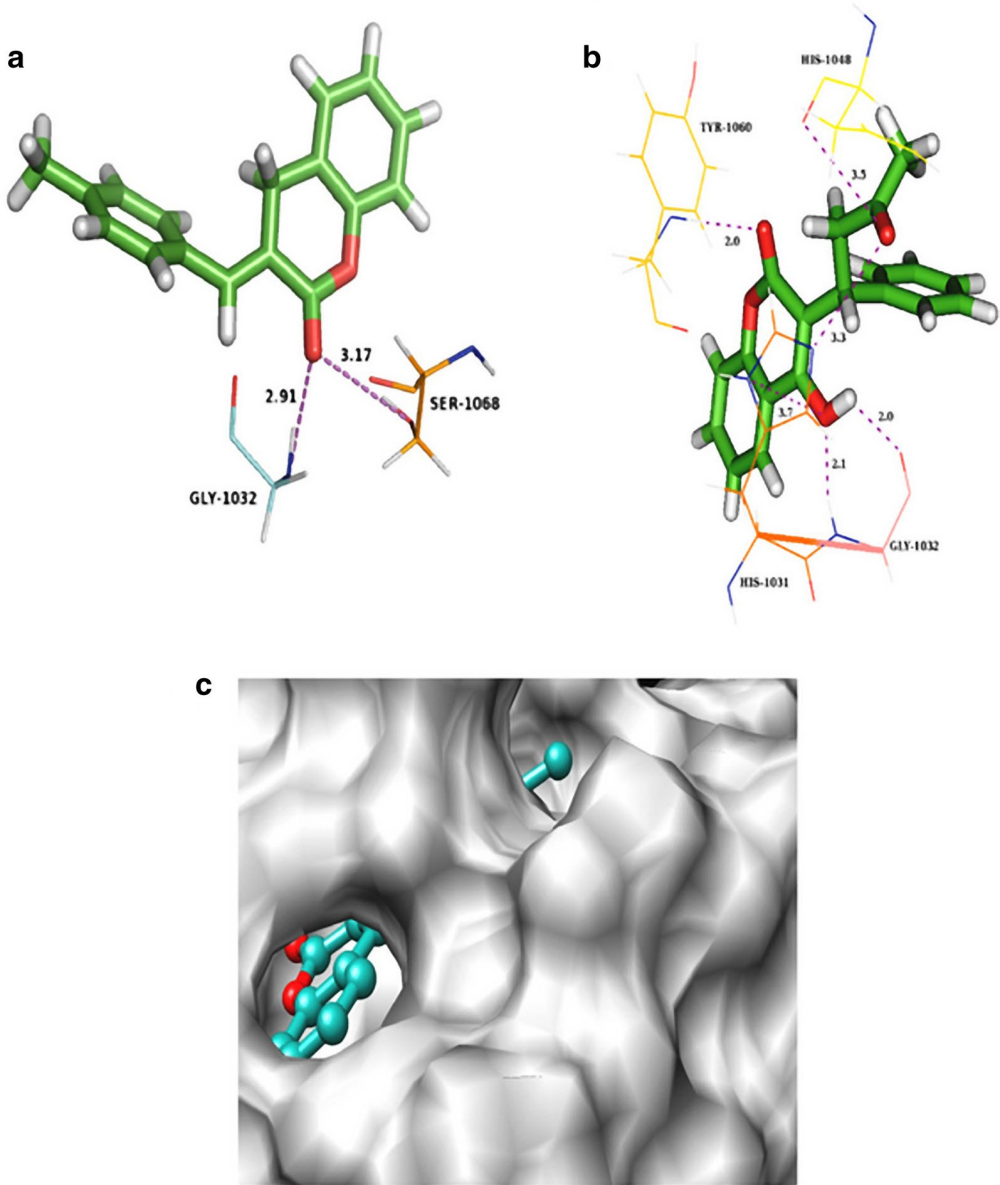
**a** MBDC interacts with the amino acid in the active site of tankyrase, **b** anticancer drug Warfarin derivative interacts with the amino acid in the active site of tankyrase, **c** surface diagram showing MBDC fit into the active site of tankyrase

Glide docking was used to study the binding orientations and affinities of MBDC with tankyrase as target protein (Fig. 7). Tankyrases are ADP-ribosyltransferases that play key roles in various cellular pathways, including the regulation of cell proliferation, and thus they are promising drug targets for the treatment of cancer [12]. The keto atom in MBDC interacts with SER1068 and GLY1032 at distances of 3.17 and 2.91 Å, respectively (Table 10). This result suggests that the MBDC binds well in the active site pocket of tankyrase and interact with the amino acid residues. These results are compared with the anti cancer drug molecule warfarin derivative. This drug molecule fits in the active site and favourable interactions are observed with the same residues. The results obtained reveals that both the molecules have comparable interactions and better docking scores.

**Table 10:** Hydrogen bond interactions of title compound and co-crystal ligand with amino acids at the active site of tankyrases

Docking score	Glide energy (kcal/mol)	Hydrogen bonding interactions		
		Donor	Acceptor	Distance (Å)
MBDC				
−10.823	−49.845	N–H[GLY1032]	O	2.91
		O–H[SER1068]	O	3.17
Warfarin				
−10.625	−55.759	NH[Tyr1060]	O	2.0
		NH[Gly1032]	O	2.1
		OH	O[Gly1032]	2.0
		OH	N[His 1031]	3.7
		N[His1031]	O	3.3
		O[His1048]	O	3.5

### Anticancer activity

**Fig. 8:**
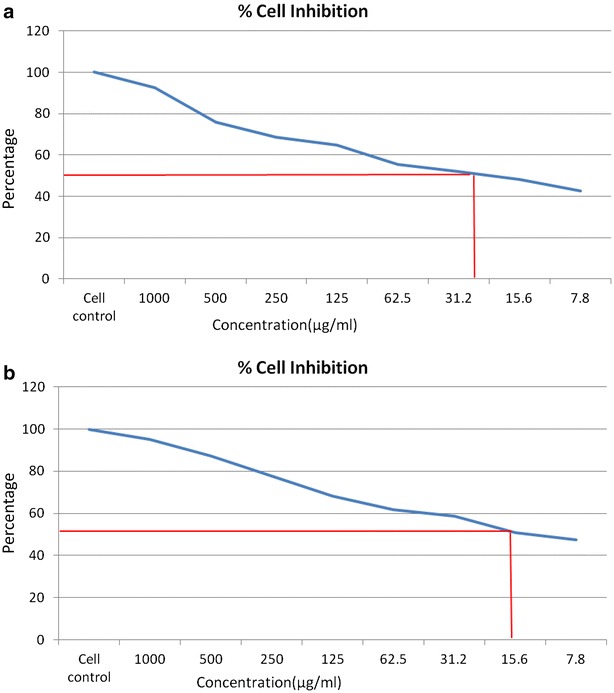
Graphical representation of MBDC molecule on **a** MCF-7 cell line and **b** HT-29 cell line

**Fig. 9:**
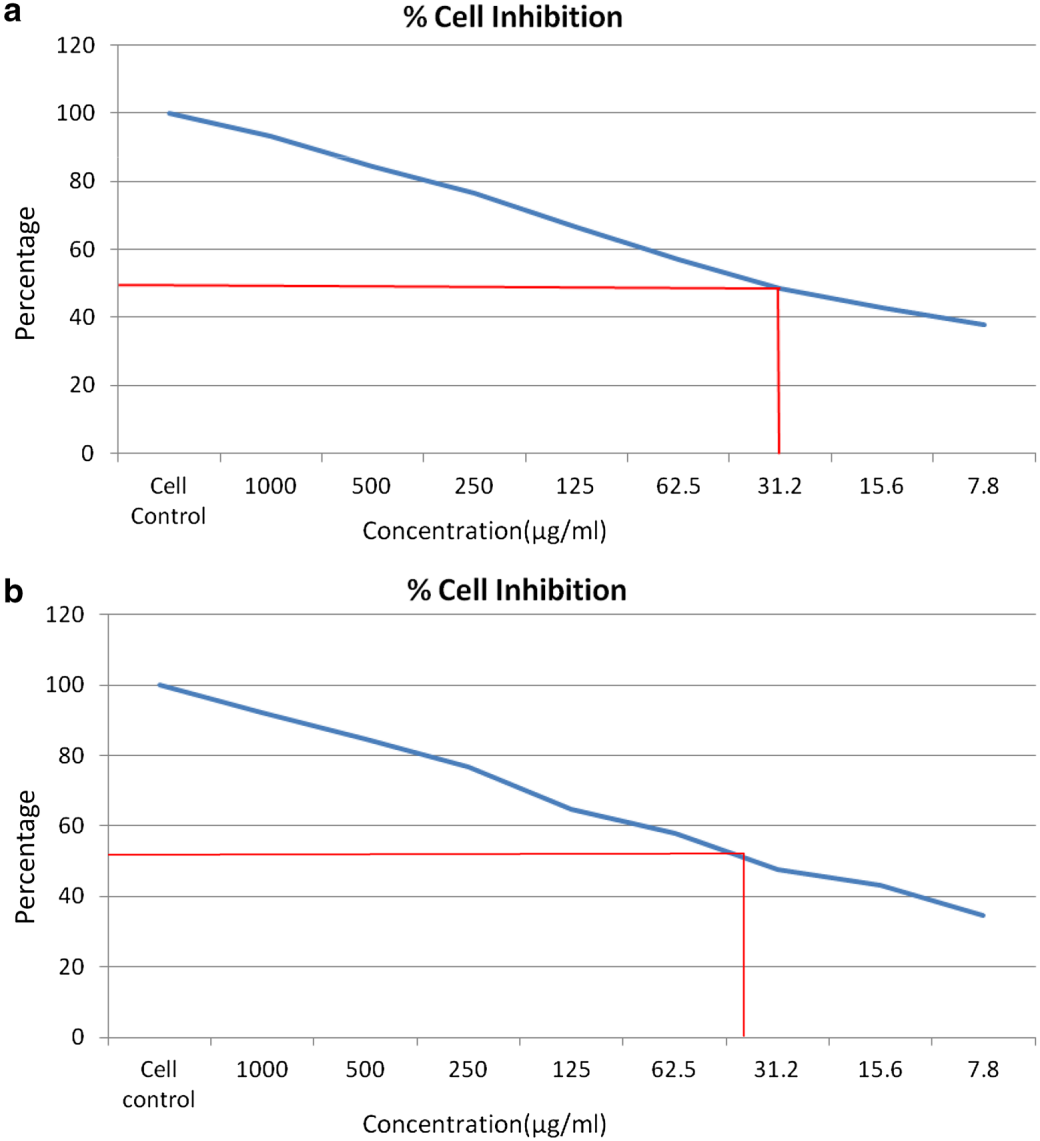
Graphical representation of Warfarin derivative on **a** MCF-7 cell line and **b** HT-29 cell line

The results of the antiproliferative activity of MBDC and Warfarin derivative against MCF-7 breast cancer and HT-29 colon cancer cell lines at different concentrations (7.8, 15.6, 31.2, 62.5, 125, 250, 500 and 1000 μg/ml) for 24 h, and cell proliferation was measured by a standard MTT assay. As shown in Figs. 8a, b and 9a, b, MCF-7 and HT-29 cells exposed to MBDC and Warfarin derivative exhibited significant cytotoxicity in the dose dependent manner after 24 h treatment. The estimated half maximal inhibitory concentration (IC 50) value for MBDC and Warfarin derivative was 15.6 and 31.2 μg/ml respectively. This enhanced cytotoxicity of MBDC in MCF-7 breast cancer and HT-29 colon cancer cell lines may be due to their efficient targeted binding and eventual uptake by the cells.

## Conclusion

The vibrational and molecular structure analysis have been performed based on the quantum mechanical approach using DFT calculations. The difference in the observed and scaled wavenumber values of most fundamentals is very small. Therefore, the assignments made using DFT theory with experimental values seem to be correct. The geometrical structure shows a little distortion due to the substitution of methyl benzylidene and chromen group in the benzene.

The chromen group substitution plays an important role with its characteristic peaks compared in both experimental and theoretical FTIR and FT-Raman spectra. The MEP map shows negative potential sites on O27 and O12 of chromen and positive potential sites on all H atoms which are responsible for electrophilic and nucleophilic attacks, respectively.

In addition, HOMO and LUMO orbitals are in agreement with MEP. The results indicate that the title compound is found to be useful to bond metallicity and inter molecular interaction. The NBO analysis explains the large delocalization of charge in the molecule. The predicted NLO properties are compared with that of urea and the title compound seems to be a good candidate of second-order NLO materials.

Molecular docking study shows that MBDC binds well in the active site of tankyrase and interact with the amino acid residues. These results are compared with the anti cancer drug molecule of warfarin derivative. The results suggest that both the molecules have comparable interactions and better docking scores. The results of the antiproliferative activity of MBDC and Warfarin derivative against MCF-7 breast cancer and HT-29 colon cancer cell lines at different concentrations exhibited significant cytotoxicity. The estimated half maximal inhibitory concentration (IC 50) value for MBDC and Warfarin derivative was 15.6 and 31.2 μg/ml, respectively. This enhanced cytotoxicity of MBDC in MCF-7 breast cancer and HT-29 colon cancer cell lines may be due to their efficient targeted binding and eventual uptake by the cells. Hence the compound MBDC may be considered as a drug molecule for cancer. The dielectric relaxation studies show the existence of molecular interactions between MBDC and alcohol. The NMR spectrum confirms the molecular structure of the compound.
